# On Maximizing the Throughput of Packet Transmission under Energy Constraints

**DOI:** 10.3390/s18072018

**Published:** 2018-06-23

**Authors:** Weiwei Wu, Guangli Dai, Yan Li, Feng Shan

**Affiliations:** 1 School of Computer Science and Engineering, Southeast University, Nanjing 211189, Jiangsu, China; shanfeng@seu.edu.cn; 2 Department of Computer Science, University of Houston, Houston, TX 77204, USA; 3 School of Information and Engineering, Nanjing University of Finance and Economics, Nanjing 210046, Jiangsu, China; yanli@njue.edu.cn

**Keywords:** energy-efficiency, rate schedule, throughput maximization, data packets, wireless data transmission, algorithm design

## Abstract

More and more Internet of Things (IoT) wireless devices have been providing ubiquitous services over the recent years. Since most of these devices are powered by batteries, a fundamental trade-off to be addressed is the depleted energy and the achieved data throughput in wireless data transmission. By exploiting the rate-adaptive capacities of wireless devices, most existing works on energy-efficient data transmission try to design rate-adaptive transmission policies to maximize the amount of transmitted data bits under the energy constraints of devices. Such solutions, however, cannot apply to scenarios where data packets have respective deadlines and only integrally transmitted data packets contribute. Thus, this paper introduces a notion of weighted throughput, which measures how much total value of data packets are successfully and integrally transmitted before their own deadlines. By designing efficient rate-adaptive transmission policies, this paper aims to make the best use of the energy and maximize the weighted throughput. What is more challenging but with practical significance, we consider the fading effect of wireless channels in both offline and online scenarios. In the offline scenario, we develop an optimal algorithm that computes the optimal solution in pseudo-polynomial time, which is the best possible solution as the problem undertaken is NP-hard. In the online scenario, we propose an efficient heuristic algorithm based on optimal properties derived for the optimal offline solution. Simulation results validate the efficiency of the proposed algorithm.

## 1. Introduction

In recent years, we have witnessed more and more Internet of Things (IoT) wireless devices providing ubiquitous services in multiple areas [1]. For instance, industrial wireless sensors may be deployed to collect various important and valuable data in time through wireless transmission; smartphones participating in crowdsensing tasks need to transmit various sensed data to a centric platform meeting the delay requirements of different requesters; wireless smart city and smart home sensors may carry out variety of surveillance and monitoring applications which impose different importance and respective deadlines for data packets in wireless communications, etc.

In such applications, data packets could have respective deadlines and diverse values. More importantly, a partially transmitted data packet may contribute nothing. For example, an incompletely transmitted data packet may be considered useless for further processing on the receiver’s side; a partially transmitted packet of sensed data may fail to provide the bottom-line quality/quantity for further data analysis. Unfortunately, most of the wireless devices in real applications are powered by capacitated batteries, which might not be able to support the transmission of all data packets in time. The incomplete transmission of data packets severely deteriorates the overall throughput that is actually achieved in wireless data transmission. Thus, we introduce a notion, named *weighted throughput*, to measure the actual achieved throughput, i.e., the total values (sum weights) of data packets that are integrally/completely transmitted within their respective deadlines. It is natural to maximize weighted throughput in wireless data transmission. Therefore, this paper addresses an important theoretical and practical question arised in such scenario,

Can we determine properly a set of data packets for delivery and design an energy-efficient rate scheduling policy to maximize the weighted throughput, i.e., the total value of successfully/integrally transmitted packets, without violating the energy constraint?

Many research efforts in prior works have been invested to address the energy-efficient tradeoffs in green wireless communications [2]. Among them, a viable solution is to exploit the rate-adaptive capacities of wireless devices and improve the efficiency of energy usage by rate scheduling. A series of works have investigated the energy-efficient rate-adaptive scheduling policies under various objectives, e.g., minimizing the energy consumption [3,4,5,6], minimizing the transmission completion time [7,8,9]. Note that all these works assume the energy is sufficient to afford the transmission of all packets, which is usually not true for battery-capacitated wireless devices. Observing this, Fu et al. [10] first design the optimal rate-adaptive scheduling policies to maximize the transmitted data bits before a given deadline with constrained energy in static channel with stable channel states. Then, Ahmed et al. [11] and Fu et al. [10] develop algorithms to maximize the transmitted data bits in fading channels under the energy constraint. Shan et al. [12] consider packets that have individual deadlines and follow FIFO service rule, and they develop the optimal algorithm to maximize the transmitted data bits. Ozel et al. [8] and Wu et al. [13] further consider energy with a certain amount can be dynamically harvested from the environment and develop offline and online competitive algorithms to maximize the data bits transmitted in fading channels. A full review on rate-adaptive scheduling is provided in Section 5. Despite all the efforts above, the throughput-maximization schedules developed in the literature are still limited to maximize the amount of transmitted data bits, which cannot be applied to the practical scenario that multiple packets are waiting for transmission while only integrally and successfully transmitted data packets contribute.

In this paper, we consider an energy-efficient rate scheduling problem over a point-to-point transmission in a fading wireless channel. We aim at designing rate schedules that maximize the weighted throughput of the battery-capacitated transmitter, subject to the integrity constraints of transmitted packets that the packets should be completely transmitted within their respective deadlines.

**Figure 1:**
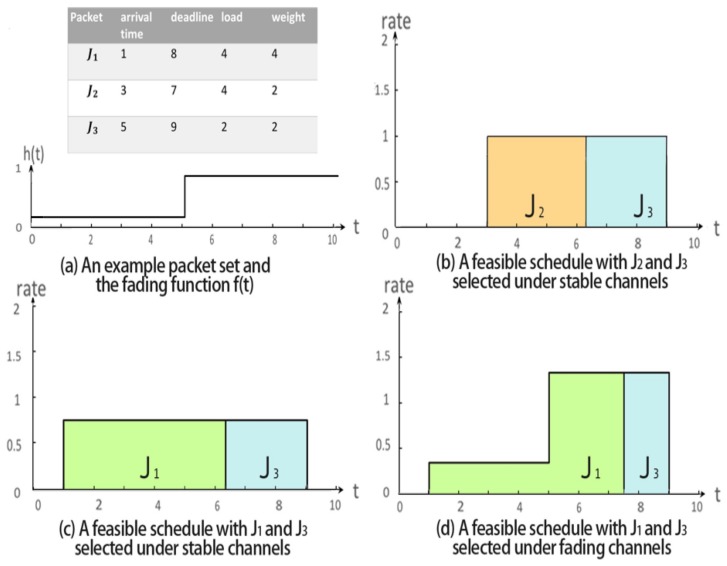
An example with three packets. The table in (**a**) shows the detailed information of the three packets and the step function in (**a**) shows the fading factors over time. (**b**–**d**) are possible rate scheduling policies under static and fading channels.

We use an example as shown in Figure 1 to illustrate the problem undertaken. Assume that there are three data packets $J_{1},J_{2}$ and $J_{3}$ waiting for delivery in a wireless channel while only an integrally transmitted packet can contribute a value/weight (which indicates its importance). Figure 1a shows the arrival time, deadline, workload and weight of each packet. Suppose that the amount of available energy for packet transmission is 30 units and we adopt a classical rate-power function. Following [14], we set the power function to be $E=\int_{t_{1}}^{t_{2}}\left(\frac{2^{2r(t)}-1}{h(t)}\right)dt$, where h(t) is the fading factor reflecting the fading effect of the channel over time, r(t) is the transmission rate to be determined, *E* denotes the consumed power, while $t_{1}$ and $t_{2}$ represent the beginning and ending time of packet transmission. Then, the transmitter cannot afford to deliver all three packets within their respective deadlines according to the results derived in [14]. As shown in Figure 1b, if the fading factor of the channel is a constant h(t)=1, then we can allocate the transmission rate r(t)=1 over time, and transmit packets $J_{2}$ and $J_{3}$ completely during (3,9] by consuming 18 units of energy, which leads to a feasible solution with a weighted throughput of 4. Given the same fading factor, if we set a fixed transmission rate 0.75 over time to successively schedule $J_{1}$ and $J_{3}$ during (1,9] as shown in Figure 1c, we can obtain another feasible solution with a weighted throughput of 6 while consuming 14.62 units of energy. Apparently, the second schedule is better than the first one. However, if the fading factor of the wireless channel varies over time as that in Figure 1a, the same rate schedule as that in Figure 1c cannot guarantee the timely transmission of $J_{1}$ and $J_{3}$ since maintaining the transmission rate at 0.75 during (1,5] would cost up to 52.56 units of energy due to the influence of the low fading factor. Thus, we need to design a proper rate schedule, e.g., the one in Figure 1d, to re-enable the schedule proposed in Figure 1c to obtain larger weighted throughput.

The above example also reveals the challenges in solving the undertaken problem,

- Since it is necessary to determine a subset of packets with high total value for delivery, it is easy to show that computing the optimal solution with maximum weighted throughput is NP-hard by reducing from the traditional knapsack problem. The difficulty mainly lies in the integral choices of packets, which differs from throughput maximization problems addressed in all prior works that aims at maximizing the data bits and admits polynomial-time optimal algorithms.
- We need to not only determine properly a subset of packets to be transmitted, but also design the corresponding optimal rate policy at the meantime. These two decisions are correlated because the chosen packets to be transmitted must admit a feasible rate schedule and vice versa.
- The conflict between the energy constraint and the integrity constraint is strengthened by the unstable fading effect of the channels, which asks for a good trade-off between energy consumption and QoS satisfaction.

In this paper, we address these challenges by developing offline and online algorithms to maximize the total value of transmitted packets under energy and integrity constraints. The contributions of this paper are as follows.

- We develop an optimal algorithm (called DLDP) with pseudo-polynomial running time to maximize the weighted throughput of packets transmitted in fading channels under energy and integrity constraints. As the problem undertaken is NP-hard, our proposed algorithm computes the best possible solution in pseudo-polynomial time. This is the first work theoretically addressing the max-throughput rate scheduling problem with the integrity constraints of transmitted data packets.
- We also develop an online heuristic algorithm by applying the proposed optimal algorithm DLDP and iteratively computing a local optimal solution. Simulation results validate its efficiency.

The rest of the paper is organized as follows. We introduce the system model and formulate the problem in Section 2. Section 3 presents an optimal pseudo-polynomial time algorithm. Section 4 provides an online heuristic algorithm and conducts the simulations to evaluate its performance. Section 5 reviews the related works. Finally, we conclude the paper in Section 6.

## 2. System Model and Problem Formulation

In this section, we will introduce the system model first, and then formulate the problem.

### 2.1. System Model

We consider a point-to-point transmission over a wireless fading channel where a transmitter needs to transmit its data packets to the receiver. Suppose that the transmitter has an amount *E* of energy available for data transmission. Assume that there are *n* data packets in the packet set $J=\{J_{1},J_{2},...,J_{n}\}$ requesting to be transmitted. For the *i*th packet $J_{i}$ in *J*, it can be described by a 4-tuple ($r_{i}$,$d_{i}$,$l_{i}$,$v_{i}$), where $r_{i}$ stands for its arrival time, $d_{i}$ refers to its deadline, $l_{i}$ is the workload to be transmitted, and $v_{i}$ is the value to be gained if $J_{i}$ is completely/successfully transmitted. Packet $J_{i}$ is only available for transmission during the time period of $\left(r_{i},d_{i}\right]$. Without loss of generality, assume $0=\min_{1\leq i\leq n}r_{i}$ and $T=\max_{1\leq i\leq n}d_{i}$. Table 1 summarizes the notations that will be used throughout the paper.

We partition the time interval into time slots, thus the arrival time $r_{i}$ and the departure time $d_{i}$ are integers. Assume that set TS is a set including all time points $r_{i}$ and $d_{i}$, for all 1≤i≤n. Thus, |TS|≤2n. We also assume that workload $l_{i}$ and the value $v_{i}$ of the packets are integers.

Denoted by function L(J) the total load $\sum_{i:J_{i}\in J}l_{i}$ and denoted by V(J) the total value $\sum_{i:J_{i}\in J}v_{i}$ of packet set *J*. We assume that the packets with earlier arrival time have no later deadlines, modeling the FIFO schedulers that follow first-in-first-out service rule [14,15], i.e., $r_{i}\leq r_{j}$ and $d_{i}\leq d_{k}$ for all i<k.

Following most of prior works [10,16], we assume that each time slot is the minimum time unit for decision, and we use a fading factor h(t) to reflect the fading effect of the channel at time slot *t*. Thus, h(t) is a piecewise constant function whose value alters only at the beginning of each time slot. During the whole time period (0,T], assuming that there are *M* pieces in h(t), we denote by $h_{k}$ the value of the *k*th piece in function h(t) that lasts $\tau_{k}$ time.

The relationship between the transmission rate and invested energy in fading channels is defined as follows. The transmission rate r(t) and the transmission power p(t) over time follows typically the concave function below [14],

(1) $$r\left(t\right)=\frac{1}{2}\log\left(1+h\left(t\right)p\left(t\right)\right),\forall t\in \left(0,T\right],$$

and correspondingly its inverse function is

(2) $$p\left(t\right)=\frac{2^{2r(t)}-1}{h(t)},\forall t\in \left(0,T\right].$$

The power allocation over time should satisfy the *energy constraint*,

(3) $$\sum_{t=1}^{T}p\left(t\right)\leq E.$$

### 2.2. Problem Formulation

Now we formulate the throughput-maximization packet transmission problem. We first define variable

(4) $$x_{i}=\left\{\begin{array}{cc} 1, & \mathrm{if}J_{i}\text{ is selected for transmission},\\ 0, & \mathrm{otherwise}. \end{array}\right.$$

We then denote $r_{it}$ as the data amount of $J_{i}$ that is transmitted in time slot *t*. Accrodingly, the transmission rate can be calculated as

(5) $$r\left(t\right)=\sum_{i=1}^{n}r_{it},\forall t\in \left(0,T\right]$$

Then, a feasible schedule should satisfy the *time constraints* and the *integrity constraints* over the transmitted packets. That is, a packet is either fully transmitted or not, and if a packet $J_{i}$ is chosen to be transmitted, all $l_{i}$ amount of its load must be completely transmitted in interval $\left(r_{i},d_{i}\right]$; Otherwise, no load of $J_{i}$ needs to be transmitted if $J_{i}$ is not chosen.

(6) $$\sum_{i=r_{i}}^{d_{i}}r_{it}=x_{i}l_{i},1\leq i\leq n.$$

**Definition 1** (MTEF problem)**.** *The weighted Max-throughput packet Transmission problem with Energy constraint under Fading channels (MTEF problem) can be formulated as:*

$$\begin{array}{cc} max\; & \sum_{i=1}^{n}x_{i}v_{i}\\ s.t.\; & Equations\;(2)–(6). \end{array}$$

Here, it is important to determine the variables $x_{i}$, i.e., the set of chosen packets in the schedule. The determination of variable p(t) is also the key to solve the MTEF problem, hence we introduce the concept of water level discussed in prior works [8],

**Definition** **2.** *The water level w(t) at time slot t is defined to be the value*

(7) $$w\left(t\right)=p\left(t\right)+\frac{1}{h(t)}$$

**Figure 2:**
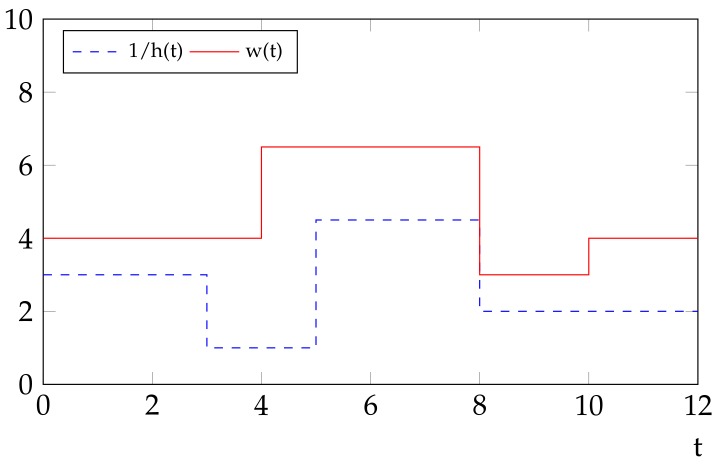
An illustration about the concept of water level w(t), which is a value of the invested power p(t) plus a constant 1/h(t), the multiplicative inverse of the fading factor .

Figure 2 illustrates the concept of the water level. In [8], energy is taken as water and the authors propose a water-filling scheme (as if the water/energy should be filled in a sink with an uneven bottom) to solve the optimization problem addressed in that paper. In intuition, if p(t) units of energy is allocated to time slot *t*, the resulting water level w(t) at that time would be p(t) plus a constant $\frac{1}{h(t)}$, the multiplicative inverse of the fading factor h(t) at time slot *t*. A further short introduction to the concept of water level can be referred to in the Appendix A.

Usually, the notion of water level is defined for ease of the discussion on the structure of the optimal solution in the literature. For example, there exists a basic property of the water level, which is given in [8],

**Lemma** **1.** *Given a schedule, suppose that the water level is $w(t_{1})$ and $w(t_{2})$ (w.l.o.g., $w\left(t_{1}\right)>w\left(t_{2}\right)$) at time slots $t_{1}$ and $t_{2}$, respectively. If the water level $w(t_{1})$ can be slightly reduced by moving partial energy allocated at time $t_{1}$ to time $t_{2}$ without violating the energy constraint and the time constraints of the packets, then such a reallocating strategy would achieve a larger amount of total transmitted workload.*

Immediately, we have

(8) $$r\left(t\right)=\frac{1}{2}\log\left(h\left(t\right)w\left(t\right)\right)$$

(9) $$p\left(t\right)=w\left(t\right)-\frac{1}{h(t)}$$

Since h(t) is a known constant at time slot *t*, it is clear that the water level w(t) can uniquely determine both the power p(t) and the rate r(t) at that time, making it another key variable we need to decide in our design.

### 2.3. Overview of Our Solutions

In this subsection, for ease of reading, we provide an overview of our solutions.

**Figure 3:**
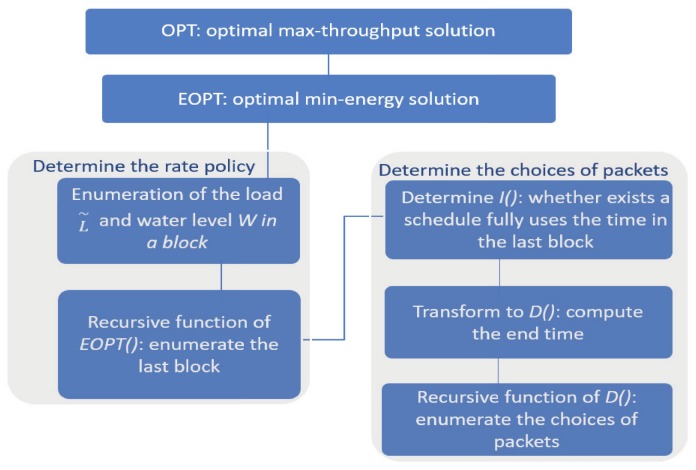
A flowchart showing the design of DLDP.

In Section 3, we will design a pseudo-polynomial time algorithm, named DLDP, to compute the optimal solution, which is the best possible as the problem will be proved to be NP-hard. The high level idea of the design is as follows. First, instead of computing the maximum achievable throughput under an energy budget (denoted as OPT) directly, we focus on a reversed version of the problem, i.e., computing the minimum energy (denoted as EOPT) needed to achieve a given total value. Then, we will develop a dynamical programming algorithm to compute the optimal solution of EOPT. Knowing that how much energy should be spent to achieve a value, we can enumerate the possible values and find how much value can be achieved given an energy budget. Figure 3 is a flowchart showing the idea of our design.

The difficulty mainly lies in computing EOPT, in which both the optimal choices of packets and its corresponding optimal rate policy should be determined. Observing this, we develop an algorithm that consists of two layers of dynamical programming process, to determine the optimal policy and the optimal choices of packets, respectively.

For the determination of optimal rate policy (Section 3.1, the first layer), as the optimal water level is pairwise linear over time, we first give a method to enumerate the possible water level in a block (during which the water level is constant), and then enumerate the shape of the last block to derive the recursive function for computing EOPT(). Note that when we try to enumerate the last block’s shape, we need a pre-condition that there exists a feasible schedule fully using the time in the last block. The verification of the binary function I() is actually related to the question whether there exists a chosen subset of packets that can fully occupy the time in the last block, which will be exactly solved in the second layer of the dynamical programming process.

For the determination of the optimal choices of packets (Section 3.2, the second layer), we first transform the computation of I() to be an end time minimization problem D(), based on which we can determine whether a chosen subset of packets can be finished exactly at the end of the last block. Then, we give a method to enumerate the choices of packets and derive the recursive function for computing D().

After deriving the recursive function of EOPT() and D(), we can implement the whole algorithm in a bottom-to-up manner to find the optimal min-energy solution EOPT as well as the optimal max-throughput solution OPT.

In Section 4, we further apply the derived optimal offline algorithm and design an online heuristic algorithm by computing a local optimal solution in each decision, which is then validated by simulations to be efficient in maximizing the weighted throughput.

## 3. Optimal Algorithm for Offline MTEF

In this section, we consider the offline MTEF problem. We first show that the problem undertaken is NP-hard.

**Lemma** **2.** MTEF problem is NP-hard.

**Proof.**  We show it by reducing from the traditional knapsack problem. In the knapsack problem, there are *n* items where the *i*-th item has value $v_{i}$ and size $c_{i}$, and we need to choose a subset of items with maximum total value without violating an capacity *C*. Given an instance of knapsack problem, we construct an instance of the MTEF problem as follows. We generate a packet set where the *i*-th packet has weight $v_{i}$ and workload $c_{i}$, and each packet has a common arrival time $r_{i}=0$ and deadline $d_{i}=T$. Moreover, we set h(t)=1 for all time 0<t≤T and the energy budget $E=T\cdot (2^{\frac{C}{T}}-1)$. Then, it is easy to see that the maximum workload that can be transmitted is $T\cdot \log(1+\frac{E}{T})=C$ by averaging all *E* amount of energy over the interval (0,T], according to the concave property of the rate-power function. Accordingly, the optimal solution of MTEF problem should transmit a subset of packets while guaranteeing that their total workload is at most *C*. Therefore, it is clear that the maximum weighted throughput is achieved if and only if the maximum total value of items chosen in the knapsack problem is found without violating the capacity constraint. This proves the NP-hardness of MTEF problem. ☐

Considering the NP-hardness of MTEF problem, we attempt to develop an optimal algorithm that runs in pseudo-polynomial time to compute the optimal solution. The high level idea of design is as follows. In order to address the energy constraint and the integrity constraint, we need to determine the rate scheduling policy and the choices of data packets for delivery. However, these two decisions are correlated with each other and difficult to be fixed simultaneously. Observing this, we attempt to deal with these two kinds of constraints separately in two layers. Thus, we propose a dynamical programming algorithm that consists of two layers of dynamical programming process, referred to as DLDP (Double Layers Dynamical Programming). The first layer enumerates to obtain the optimal distribution of power/rate, mainly dealing with the energy constraint, while the second layer mainly deals with the integrity constraints, i.e., the choices of packets to be transmitted. With correct packets selected by the second layer, the first layer returns the optimal rate schedule with the maximum value gained by transmitting the packets under the energy constraint.

Furthermore, instead of a direct study of the maximum throughput achievable (OPT), we compute the minimum energy consumption needed to achieve a certain total value as follows. Denoted by $EOPT(t_{s},t_{e},S,V)$ the minimum energy consumption for transmitting a subset of packets in *S* with the total value added up at least *V* in a specific time period $\left(t_{s},t_{e}\right]$. Knowing that how much energy should be spent to achieve a value, we can find the maximum value OPT by enumerating the possible values and checking whether the consumed energy satisfies the energy constraint. Thus, we will mainly focus on the computation of EOPT().

We will introduce the two layers of the designed dynamical programming process in Section 3.1 and Section 3.2 first, and then combine them together in Section 3.3 to obtain the final algorithm and validate its optimality.

### 3.1. First Layer of DLDP

In this subsection, we introduce the first layer of DLDP designed to determine the optimal rate policy.

Since the optimal rate policy can be determined once the water level at each time slot is found, we start by introducing a basic lemma about the water level [14].

**Lemma** **3.** For any optimal schedules, if the water level w(t) increases or decreases at time slot t, then t must be an arrival time or a deadline of one or more packets, i.e., t∈TS.

According to the lemma above, it is obvious that water level w(t) of the optimal schedule is a piecewise constant function that varies only at time t∈TS. Accordingly, we assume that a block in the optimal solution is the longest interval in which the optimal solution keeps at a constant water level. Immediately, we know that the optimal solution is composed of blocks, each with a certain constant water level.

Observing this, we attempt to figure out what does the shape of the optimal solution look like in terms of blocks. Our idea in designing the dynamical programming process here is to enumerate the possible water level of the last block in the optimal solution. Once we found the last block, we can combine it with the solution computed for the remaining intervals (which could be taken as a sub-problem), and then return as the final solution. To achieve it, we need to address two issues, (1) how to enumerate the water level in the last block of the optimal solution considering the possible water level could be any real value, and (2) how to combine the results and return as the final solution.

First, we introduce the method on enumerating the water level of the last block in the optimal solution. Although the water level could be any real value, if the water level stays steady at a certain value w(t)=W in a specific period in the optimal solution, say $\left(t_{1},t_{2}\right]$ with $t_{1},t_{2}\in TS$, we show that it is possible to restrict it in a candidate set with a finite size. Obviously, the start time $t_{1}$ and end time $t_{2}$ of the block can be enumerated within $O(n^{2})$ time since $t_{1},t_{2}\in TS$. Furthermore, we have

(10) $$\begin{array}{cc} \tilde{L} & =\int_{t_{1}}^{t_{2}}r\left(t\right)dt\\ & =\frac{1}{2}\int_{t_{1}}^{t_{2}}\log\left(h\left(t\right)W\right)dt \end{array}$$

where $\tilde{L}$ is the total load/amount of data transmitted in that interval. Recall that h(t) is piecewise constant. To simplify the calculation, we assume that during $\left(t_{1},t_{2}\right]$, the function h(t) is composed of *m* pieces, from the *a*th piece to the (a+m−1)th piece. Thus, Equation (10) can be rewritten as,

(11) $$\begin{array}{c} \begin{array}{c} \tilde{L}=\frac{1}{2}\sum_{k=a}^{a+m-1}\log\left(h_{k}W\right)\tau_{k} \end{array} \end{array}$$

According to the property above, once the total load transmitted in that block is fixed, we can reversely determine the water level therein. Observing this, we only need to show that the total load transmitted in that interval in the optimal solution can be enumerated. Therefore, in the following discussion, we prove that if the interval $\left(t_{1},t_{2}\right]$ is chosen correctly, the total load transmitted in the optimal solution is the total workload of a certain subset of the input packets.

**Lemma** **4.** In an optimal schedule, if $t_{1}$ and $t_{2}$ are both time points at which the water level changes, the total load transmitted during $\left(t_{1},t_{2}\right]$ must be the total workload of a certain subset of the input packet set.

**Proof.**  We use $J_{\left(t_{1},t_{2}\right]}$ to denote the set of packets that are transmitted during $\left(t_{1},t_{2}\right]$. We only need to show that no packet in $J_{\left(t_{1},t_{2}\right]}$ is partially transmitted in interval $\left(t_{1},t_{2}\right]$. Suppose a certain packet $J_{i}\in J_{\left(t_{1},t_{2}\right]}$ is partially transmitted there. W.l.o.g., suppose it starts transmission at $t^{\prime }$ where $t^{\prime }\leq t_{1}$ (the proof for the case that it ends transmission after $t_{2}$ is similar), and it also transmits at a time $\hat{t}\in \left(t_{1},t_{2}\right]$. This implies $r_{i}\leq t^{\prime }\leq t_{1}<d_{i}$. According to the prior condition, the water level changes at $t_{1}$. W.l.o.g. we assume that the water level $w\left(\hat{t}\right)>w\left(t_{1}\right)$. Thus, applying Lemma 1, we can slightly reduce the water level by moving partial workload of $J_{i}$ at time $\hat{t}$ to time $t_{1}$. This would reduce the energy consumption by Lemma 1, which contradicts the optimality of the schedule. Hence, all the packets in $J_{\left(t_{1},t_{2}\right]}$ are all completely transmitted during $\left(t_{1},t_{2}\right]$. This proves the lemma. ☐

Since the total workload of any subset in *J* is an integer no greater than L(J), we can enumerate the load transmitted in interval $\left(t_{1},t_{2}\right]$ in O(L(J)) time. Therefore, given a total load $\tilde{L}$ and a specific period $\left(t_{1},t_{2}\right]$, we can solve Equation (11) and get the value *W*, which will be denoted by function $GW(\tilde{L},t_{1},t_{2})$ for ease of discussion. That is,

(12) $$W=GW\left(\tilde{L},t_{1},t_{2}\right)={\left(\frac{2^{2\tilde{L}}}{\prod_{k=a}^{a+m-1}h_{k}^{\tau_{k}}}\right)}^{\frac{1}{\sum_{k=a}^{a+m-1}\tau_{k}}}$$

Now we have a fierce relationship between *W* and $\tilde{L}$. Note that although $t_{1},t_{2}$ has not shown up in the right side of Equation (12). They play a vital role since they provide a certain period which determines the values $h_{k}$ in that period. By enumerating $t_{1}$, $t_{2}$ and $\tilde{L}$, we could figure out the shape of the last block in the optimal schedule.

The analysis above has provided a method to fix the last block of the optimal solution. Now, we are ready to derive the recursion function of $EOPT(t_{s},t_{e},S,V)$. We need to fix the remaining intervals excluding the last block and return the final solution using dynamical programming process. Note that there is no need to make full use of the time in $\left(t_{s},t_{e}\right]$ during the transmission.

**Figure 4:**
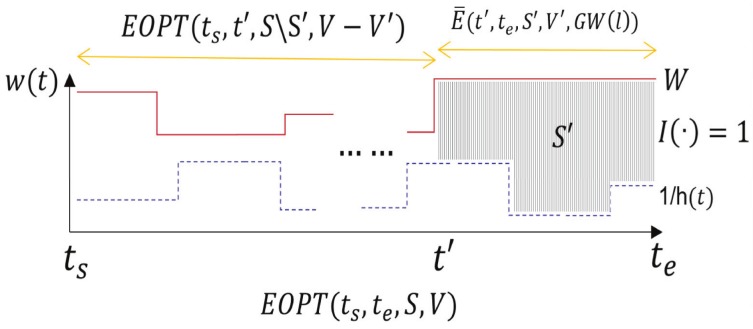
An example showing the recursive function of EOPT(·).

Figure 4 demonstrates an example showing the recursive function of EOPT(·). Given a general interval $\left(t_{s},t_{e}\right]$, suppose that the optimal solution keeps at a steady water level *W* in the last block, say $\left(t^{\prime },t_{e}\right]$. Assume that the total value achieved therein is $V^{\prime }$ and the corresponding transmitted packets are chosen from a set $S^{\prime }\subseteq S$. Then, the energy consumption in $\left(t^{\prime },t_{e}\right]$ would be $\int_{t^{\prime }}^{t_{e}}W-\frac{1}{h(t)}dt$ if the time in that period is *fully* used to transmit a subset of packets from set $S^{\prime }$. Thus, as a trick, we use a binary function $I(t^{\prime },t_{e},S^{\prime },V^{\prime },W)$ (to be determined later) to identify whether there exists a feasible schedule that keeps at a water level *W* in $\left(t^{\prime },t_{e}\right]$ and transmits a subset of packets from $S^{\prime }$ with the total value $V^{\prime }$ achieved by fully occupying the time in that period. Accordingly, the energy consumption in the remaining sub-interval $\left(t_{s},t^{\prime }\right]$ can be returned recursively by function $EOPT(t_{s},t^{\prime },S\backslash S^{\prime },V-V^{\prime })$, which transmits packets from the remaining set $S\backslash S^{\prime }$ while achieving a total value $V-V^{\prime }$. Thus, the total energy consumption in $\left(t_{s},t_{e}\right]$ could be returned by combining these two parts. Therefore, if I(·)=1, then the total energy consumption $EOPT(t_{s},t_{e},S,V)$ in $\left(t_{s},t_{e}\right]$ equals to that of $EOPT(t_{s},t^{\prime },S\backslash S^{\prime },V-V^{\prime })$ plus $\int_{t^{\prime }}^{t_{e}}W-\frac{1}{h(t)}dt$. Otherwise, the value is set with $EOPT(t_{s},t_{e},S,V)=\infty$ when I(·)=0. To simplify the presentation, we use $\bar{E}\left(t^{\prime },t_{e},S^{\prime },V^{\prime },W\right)$ to return value $\int_{t^{\prime }}^{t_{e}}W-\frac{1}{h(t)}dt$ if I(·)=1 and value ∞ otherwise. That is,

$$\begin{array}{c} \bar{E}\left(t^{\prime },t_{e},S^{\prime },V^{\prime },W\right)=\left\{\begin{array}{c} \int_{t^{\prime }}^{t_{e}}W-\frac{1}{h(t)}dt,\;\mathrm{if}\;I\left(\cdot \right)=1\\ \infty ,\;\;\;\;\;\;\mathrm{otherwise} \end{array}\right. \end{array}$$

Note that in the analysis above, the solution to function I(·) is related to the question that which subset of packets should be chosen to be transmitted. We will leave the discussion on such solution in the next subsection.

It remains to show how to divide a packet set *S* into two sets $S^{\prime }$ and $S\backslash S^{\prime }$ during the recursive process above, considering this may cost exponential computation time. Hence, we observe what is the order of packet transmission in the optimal schedule, which is stated in the following lemma.

**Lemma** **5.** Among optimal schedules, there always exists a schedule where all packets are transmitted without preemption and the transmission follows the FIFO rule.

**Proof.**  Assume that in the optimal schedule, there are two intervals $\left(a_{1},a_{2}\right]$ and $\left(a_{2},a_{3}\right]$ with $a_{1}<a_{2}<a_{3}$. Suppose that there are two packets $J_{i}$, $J_{k}$ with i>k, where a segment of $J_{i}$ is transmitted during $\left(a_{1},a_{2}\right]$ and a segment of $J_{k}$ is transmitted during $\left(a_{2},a_{3}\right]$. Then, when swapping the transmission order of the packets in these two segments by transmitting packet $J_{k}$ earlier, though the transmission time of these two packets may change, the transmission process of these two packets will still start at $a_{1}$ and end by $a_{3}$. No deadline (or arrival time) constraints of the packets will be violated, because $d_{k}\leq d_{i}$ and $r_{k}\leq r_{i}$ stands when i>k. Moreover, swapping the transmitting order will not influence the transmission of segments before or after them. Hence, this proves the feasibility of the swap of two adjacent segments if the packet transmitted in the first segment has later deadline than the second.

With the feasibility of swapping operation above, it is not a harsh task to gather all the segments of the same packet to adjacent time periods by swapping, so that each packet is transmitted in a single piece. Therefore, for any optimal schedule, we can utilize the swapping operation to transform it into another optimal schedule without preemption. Similarly, we can further transform it into a schedule following the FIFO rule. This completes the proof. ☐

**Remark** **1.** Recall that the water level function w(t) directly determines the power function p(t) and the rate function r(t). The lemma above implies that once all the variants w(t) and $x_{i}$ (indicating which packets are selected) are determined, then by transmitting the selected packets in FIFO order, we can decide how much workload of a packet should be transmitted at any time, i.e., the variables $r_{it}$. This allows us to focus on the discussion about how to determine the water level as stated above and the packet selection in the next subsection.

We use $J_{a,b}$ to denote the set of packets $S=\{J_{i}|a\leq i\leq b\}$. Consider the full set $S=\left\{J_{i}|1\leq i\leq n\right\}=J_{1,n}$ first. Lemma 5 states that the transmission of the optimal schedule follows FIFO rule, thus the first $k^{\prime }$ (an index to be enumerated) packets with lower index in set *S* is transmitted before other packets. This allows us to partition packet set *S* into two separate parts by allocating the first $k^{\prime }$ packets into the first part ($J_{1,k^{\prime }}=S\backslash S^{\prime }$) and allocating the others into the second part ($S^{\prime }=J_{k^{\prime }+1,n}$). Such partition is unique once $k^{\prime }$ is fixed and will not affect the optimality in calculating EOPT(·). For the general case that $S=J_{1,k}$, then we have $S^{\prime }=J_{k^{\prime }+1,k}$ and $S\backslash S^{\prime }=J_{1,k^{\prime }}$.

Therefore, by numerating the possible values of $k^{\prime }$ and the possible values of *W*, we have the final recursion function for EOPT(·), as concluded in Theorem 1. Note that to shorten the equation, we present $GW(l,t^{\prime },t_{e})$ as GW(l).

**Theorem** **1.** $$\begin{array}{c} EOPT\left(t_{s},t_{e},J_{1,k},V\right)=\min_{t^{\prime },k^{\prime },V^{\prime },l}EOPT\left(t_{s},t^{\prime },J_{1,k^{\prime }},V-V^{\prime }\right\})+\bar{E}\left(t^{\prime },t_{e},J_{k^{\prime }+1,k},V^{\prime },GW\left(l\right)\right). \end{array}$$

After deriving the recursive function for computing EOPT(·), we present its detailed implementation in Algorithm 1. In the algorithm, we introduce a three-dimensional matrix Eopt. We use Eopt[i][j][k] to denote the minimum energy needed to accomplish packets selected from the set made up of the first *i* packets which attains a total value *j* before time TS[k]. The recursion is then implemented in a bottom-to-top manner.

This completes the design for the first layer of DLDP, which determines the water level and correspondingly the rate policy in the optimal solution. Note that it remains to determine the function I(·), which is related to the optimal choices of the packets to be transmitted and will be solved in the second layer of the dynamical programming process in DLDP.

### 3.2. Second Layer of DLDP

After fixing the rate policy, we now determine the optimal integral choices of packets to be transmitted. The answer to it is related to the computation of $I(t^{\prime },t_{e},S^{\prime },V^{\prime },W)$, which is needed in computing $\bar{E}\left(t^{\prime },t_{e},J_{k^{\prime }+1,k},W\right)$ in Theorem 1, where $S^{\prime }=J_{k^{\prime }+1,k}$. For ease of presentation, we write $S^{\prime }=J_{p,q}=\left\{J_{i}|p\leq i\leq q\right\}$ by replacing the indexes of $S^{\prime }$ in the following discussions.

Recall that the binary function I(·) is used to identify whether there exists a feasible schedule that transmits a subset of packets in $S^{\prime }$ at a steady water level *W* while achieving a total value $V^{\prime }$ in $\left(t^{\prime },t_{e}\right]$. In order to give the answer to I(·), we introduce a new function $D(t^{\prime },S^{\prime },V^{\prime },W)$, which denotes the minimum time needed to transmit a subset of packets from $S^{\prime }$ while achieving a total value $V^{\prime }$ with a steady water level *W* after time $t^{\prime }$. Thus, if there exists a feasible schedule transmitting a subset of packets from $S^{\prime }$ with the total value $V^{\prime }$ achieved by fully occupying the time in the period $\left(t^{\prime },t_{e}\right]$, then we have $D\left(\cdot \right)=t_{e}$. It is easy to see that I(·)=1 if and only if $D\left(\cdot \right)=t_{e}$, and furthermore, I(·)=0 if and only if $D\left(\cdot \right)\neq t_{e}$. Therefore, we have transformed the computation of I(·) to the end time minimization problem in computing D(·).

Now we focus on the computation of D(·). Since partially transmitted packets contribute nothing, there must exist an optimal schedule that does not partially transmit any packet for the consideration of energy efficiency. Moreover, according to Lemma 5, the chosen packets are transmitted in FIFO order. Thus, we only need to discuss about the possible schedules that transmit packets in FIFO order and does not partially transmit any packet in $\left(t^{\prime },t_{e}\right]$. Based on these observations, we introduce the other layer of dynamical programming process and compute D(·) by considering the packets one by one and distinguishing the cases whether a packet is selected or not.

To simplify the expression, we write $\bar{D}\left(p,q,V\right)=D\left(t^{\prime },\left\{J_{i}|p\leq i\leq q\right\},V,W\right)$ where $t^{\prime }$ and *W* are not shown in the input of $\bar{D}\left(\cdot \right)$. This allows us to focus on the calculation of $\bar{D}\left(p,q,V\right)$. Thus, all the rate schedule mentioned below starts at time $t^{\prime }$, and during the transmission, the water level stays at a constant value *W* by default.
**Algorithm 1**$EOPT(t_{s},t_{e},J,V)$**Input:**$t_{s}$,$t_{e}$,*J*,*V* **Output:**minEC1:Use sorted array TS to store all time points of the arrival time and deadline of packets in *J*.2:Let Eopt be a three-dimension matrix with size ((|J|+1)·(V+1)·(|TS|+1)) that is initiated with value MAX_VALUE=∞.3:**for**i=1**to**|J|, j=0**to***V*, k=1**to**|TS|**do**4: **if** j=0 **then**5:  Eopt[i][j][k]←06:  **continue**7: **end if**8: minE←MAX_VALUE9: **for** y=0 **to** *i* **do**
10:  $SB\leftarrow \{J_{i}|1\leq i\leq y\}$11:  $SA\leftarrow \{J_{i}|y<i\leq |J|\}$12:  **for** v=0 **to** *j*, t=1 **to** *k* **do**13:   z←MAX_VALUE14:   **for** l=0 **to** L(SA) **do**15:    **if** I(TS[t],SA,v,GW(l))=1 **then**16:     $z\leftarrow \min\{\int_{TS[t]}^{TS[k]}(GW\left(l\right)-\frac{1}{h(t)})dt,z\}$17:    **end if**18:   **end for**19:   **if** y>0 **then**20:    tempWE←Eopt[y][j−v][TS[t]]21:   **else**22:    tempWE←023:   **end if**24:   minE←min{minE,z+tempWE}25:  **end for**26: **end for**27: Eopt[i][j][k]←minE28:**end for**29:**return**Eopt[|J|][V][|TS|]

Denoted by $T(a,J_{k},W)$ the end time of transmitting $J_{k}$ when it is transmitted immediately after time *a* while the water level keeps steady at *W*. Note that such a value can be directly calculated given all known values of $l_{k}$, h(t) and *W* by solving the unknown variant *x* in the equation $\int_{a}^{x}\log\left(h\left(t\right)W\right)dt=l_{k}$. Note that if the calculated end time is larger than $d_{k}$, then we would set $T(a,J_{k},W)=+\infty$. In the following discussion, we would simplify $T(a,J_{k},W)$ as $T(a,J_{k})$ if no ambiguity arises.

Based on this definition, we can derive a recursive function of $\bar{D}\left(p,q,V\right)$ by distinguishing the cases whether $J_{q}$ is chosen to transmit or not.

**Theorem** **2.** $$\begin{array}{c} \bar{D}\left(p,q,V\right)=\min\left\{\begin{array}{c} T(\max\{r_{q},\bar{D}\left(p,q-1,V-v_{q}\right)\},J_{q})\\ \bar{D}\left(p,q-1,V\right) \end{array}\right. \end{array}$$

**Proof.**  Note that the variants $\left\{x_{p},x_{p+1},...,x_{q}\right\}$ determine the packets chosen to be transmitted in $\bar{D}\left(p,q,V\right)$. Recall Lemma 5 that there exists an optimal schedule transmitting the packets with FIFO order without preemption. Moreover, the optimal schedule would not partially transmit any packet for energy efficiency. Hence, we just integrally transmit the chosen packets in FIFO order. With such pre-condition, we will call $\bar{D}\left(p,q-1,\cdot \right)$ to deal with the packets excluding the last one $J_{q}$. To derive the recursive function for D(·), we distinguish two cases that $x_{q}$ is 0 or 1.

**Case 1** ($x_{q}=1$): On one hand, if $T\left(\max\{r_{q},\bar{D}\left(p,q-1,V-v_{q}\right)\},J_{q}\right)<\bar{D}\left(p,q,V\right)$, there exists a value $T(\max\{r_{q},DP\left(p,q-1,V-v_{q}\right)\},J_{q})$ that is smaller than $\bar{D}\left(p,q,V\right)$. Then the end time of $\bar{D}\left(p,q,V\right)$ can be reduced by scheduling $J_{q}$ right after time $\max\{r_{q},D\left(p,q-1,V-v_{q}\right)\}$, contradicting the optimality of $\bar{D}\left(p,q,V\right)$.

On the other hand, if $T\left(\max\{r_{q},\bar{D}\left(p,q-1,V-v_{q}\right)\},J_{q}\right)>\bar{D}\left(p,q,V\right)$, then the end time used for scheduling packets $\left\{J_{i}|p\leq i\leq q-1\right\}$ while achieving a value $V-v_{q}$ should be at least $\bar{D}\left(p,q-1,V-v_{q}\right)$. As a result, the end time used for scheduling packets $\left\{J_{i}|p\leq i\leq q\right\}$ while achieving a value *V* should be at least $T(\max\{r_{q},\bar{D}\left(p,q-1,V-v_{q}\right)\}$. This implies $\bar{D}\left(p,q,V\right)\geq T(\max\{r_{q},\bar{D}\left(p,q-1,V-v_{q}\right)\}>\bar{D}\left(p,q,V\right)$, leading to a contradiction again. Hence, if $x_{q}=1$, then $\bar{D}\left(p,q,V,\right)=T\left(\max\{r_{q},\bar{D}\left(p,q-1,V-v_{q}\right)\},J_{q}\right)$.

**Case 2** ($x_{q}=0$): On one hand, if $\bar{D}\left(p,q-1,V\right)>\bar{D}\left(p,q,V\right)$, then the absence of $J_{q}$ in the optimal schedule of $\bar{D}\left(p,q-1,V\right)$ incurs a larger end time than $\bar{D}\left(p,q,V\right)$, which is impossible.

On the other hand, if $\bar{D}\left(p,q-1,V\right)<\bar{D}\left(p,q,V\right)$, then the value of $\bar{D}\left(p,q,V\right)$ can be reduced to be $\bar{D}\left(p,q-1,V\right)$ since $x_{q}=0$. Therefore, if $x_{q}=0$, then $\bar{D}\left(p,q,V\right)=\bar{D}\left(p,q-1,V\right)$.

Based on the discussion above, we have the recursive function. ☐

After deriving the recursive function for computing D(·), we present its detailed implementation in Algorithm 2.
**Algorithm 2**$D(t_{s},J,V,W)$**Input:**$t_{s}$, *J*,*V*,*W* **Output:**endTime1:Let $\tilde{D}$ be a two-dimension matrix with size ((|J|+1)·(V+1)) that is initiated with value MAX_VALUE.2:**for**$j=v_{1}$**to***V***do**3: $\tilde{D}\left[1\right]\left[j\right]\leftarrow T\left(\max\left\{r_{1},t_{s}\right\},J_{1}\right)$4:**end for**5:**for**i=2**to**|J|**do**6: **for** j=0 **to** *V* **do**7:  $minT\leftarrow \tilde{D}\left[i-1\right]\left[j\right]$8:  **if** $v_{i}\leq j$ **then**9:   $temp\leftarrow T(\max\left(\tilde{D}\left[i-1\right]\left[j-v_{i}\right],r_{i}\right),J_{i})$10:   **if** temp<minT **and** $temp\leq d_{i}$ **then**11:    minT←temp12:   **end if**13:  **end if**14:  $\tilde{D}\left[i\right]\left[j\right]\leftarrow minT$15: **end for**16:**end for**17:**return**$\tilde{D}\left[|J|\right]\left[V\right]$

### 3.3. Merging Together: The Optimal Algorithm

Combining the results above, we have the final optimal algorithm to compute the minimum energy consumption EOPT(·) needed to achieve a certain total value *V*. Reversely, the optimal weighted throughput (denoted as OPT) under energy budget *E* can then be acquired by testing the value *V* and searching the maximum one that satisfies the energy constraint. That is,

(13) $$\begin{array}{cc} OPT & =\max_{0\leq V^{\prime }\leq V\left(J\right),EOPT\left(0,T,J,V^{\prime }\right)\leq E}\left\{V^{\prime }\right\} \end{array}$$

Finally, we have the final algorithm DLDP (presented in Algorithm 3) to compute the optimal solution for the MTEF problem. The following theorem concludes the optimality of DLDP and its time complexity.
**Algorithm 3** DLDP(Double Layers Dynamical Programming)**Input:***J*, *E* **Output:**OPT1:$totalValue\leftarrow \sum_{i=1}^{\left|J\right|}v_{i}$2:$t_{s}\leftarrow r_{1}$3:$t_{e}\leftarrow d_{\left|J\right|}$4:**for**j=totalValue**to** 0 **do**5: **if** $EOPT(t_{s},t_{e},J,j)\leq E$ **then**6:  **return** *j*7: **end if**8:**end for**9:**return** 0

**Theorem** **3.** Algorithm DLDP computes the optimal solution in $O(n^{4}LV^{2}M)$ time.

**Proof.**  The optimality of the algorithm directly follows by the analysis and the correctness of the recursion functions that are derived above. To show the time complexity of the overall algorithm, we analyze the running time of each sub-procedure. In computing D(·), the input includes an index of packets in *J*, a start time, a given water level *W* and a value *V*. Thus, the size for matrix *D* should be $O(n^{3}LV)$ where *L* is the total load and *V* is the total value of input packet set *J*. For each unit of matrix *D*, it takes O(nVM) to calculate the result. Hence the running time of computing D(·) is $O(n^{4}LVM)$. In computing EOPT(·), it costs O(1) time when EOPT(·) calls D(·) if D(·) is pre-computed. The matrix for computing EOPT(·) should have a size $n^{2}V$. To calculate each unit of the matrix, another $O(n^{2}VLM)$ time will be needed in Algorithm 1. Therefore, the time complexity for computing EOPT(·) is $O(n^{4}LV^{2}M)$. Combining these results, the final time complexity of DLDP is $O(n^{4}LV^{2}M)$. ☐

## 4. Online Heuristic Algorithm for MTEF

In the previous section, we have developed an optimal algorithm with pseudo-polynomial running time for the offline MTEF problem. We note that DLDP returns the best possible solution as the problem undertaken is NP-hard. In this section, we consider the online scenario of the MTEF problem. We will propose an online heuristic algorithm and validate its efficiency by simulations.

### 4.1. Online Heuristic Algorithm

In this subsection, we develop an online algorithm for the online MTEF problem. We note that the optimal algorithm DLDP can return the optimal solution efficiently. Thus, we will adopt it as a building block to compute a local optimal solution and accordingly design an efficient online schedule.

The difficulty in coping with the online problem lies mainly on the absence of future information and the irreversible property of operations over time. On one hand, we need to determine how much energy should be allocated as time goes by. On the other hand, we should decide which subset of packets are chosen to be transmitted. By the concave property of the rate-power function, a high-rate transmission of a packet is more energy consuming than a low-rate transmission that takes a longer time to transmit. Thus, the optimal schedule prefers to start transmission of a packet early from its arrival time until its deadline. The later the transmission of the packet starts, the more energy will be consumed on it. Yet if the transmission starts right at the time packets arrive, it may have to abandon some partially transmitted packets when another packet benefiting more comes afterwards due to the energy constraint. However, this is a potential loss since partial transmission contributes nothing.

Observing this, we introduce the concept of “hesitation” in order to provide an opportunity for the schedule to gather more information before making a decision. Specifically, we wait for a certain number of time slots before another decision is made. Furthermore, we adopt the offline optimal algorithm DLDP as a building block to determine how much energy should be allocated in each decision.

The detailed design of the online algorithm (named HST) is presented in Algorithm 4. When the last round of transmission is done, it will wait for *u* more time slots to gather a set *S* of candidate packets for the next round of transmission. One key issue remains to be addressed is to determine how much energy should be distributed into different rounds. In order to provide a critical criterion, we define the local energy efficiency as the value-per-energy $LEE=\frac{DLDP(S,e)}{e}$, where DLDP(S,e) is the maximum value returned by Algorithm DLDP with an input of packet set *S* and energy budget *e*. Then, we find the most efficient one $\bar{E}=\arg\max_{e}\frac{DLDP(S,e)}{e}$ where *e* is treated as integer numbers considering the enumeration of a real value *e* is impractical. Finally, we assign $\bar{E}$ units of energy in the current round of transmission and the algorithm proceeds until no more energy available.
**Algorithm 4** HST (u)**Input:**J,E,u **Output:***V*1:Let $T_{r}$ be the beginning time of round *r* and $C_{r}$ be the end time of the local schedule made in round *r*. Initially r=0 and $T_{0}=C_{0}=0$.2:**for**i=1**to**|J|**and**E>0**do**3: $S\leftarrow \{J_{i}|T_{r}<r_{i}\leq C_{r}+u\}$4: Modify all arrival time of packets in *S* to be $C_{r}+u$.5: $\bar{E}=\arg\max_{e:0<e\leq E}\frac{DLDP(S,e)}{e}$6: Start a new round r+1 by transmitting packets according to the local schedule made by $DLDP(S,\bar{E})$.7: $V\leftarrow V+DLDP(S,\bar{E})$8: Update $E\leftarrow E-\bar{E}$.9: Update $T_{r+1}\leftarrow C_{r}+u$10: Update $C_{r+1}$ to be the end time of the local schedule made by $DLDP(S,\bar{E})$.11: Update r←r+112:**end for**13:**return***V*

The key design above is to apply the proposed optimal algorithm DLDP and compute a local optimal solution for the arrived tasks that remain alive. Note that when it runs with the general input of non-FIFO tasks, a local optimal solution can still be computed by DLDP for the arrived tasks since they follow FIFO order. Thus, HST is actually applicable for non-FIFO tasks.

### 4.2. Simulations

In this subsection, we conduct simulations to validate the performance of the proposed online algorithm HST. We implement our simulations by Python 2.7. In the simulations, considering that no prior works have addressed the problem same to our setting, we will compare our online algorithm with two baselines and an algorithm refined from [12], which are listed as follows.

- **Greedy**, which attempts to transmit all packets from their arrival time to their deadlines without waiting. For each packet that arrives, Greedy will immediately transmit the packet, and increase the transmission rate to the lowest needed in terms of deadlines based on the current schedule. This suggests that Greedy does not abandon packets unless there is not enough energy left.
- **GreedyDrop**, which is a schedule that improves Greedy by actively dropping packets on their arrivals if they have relatively low values. In detail, it abandons a task on its arrvial if its values is smaller than the median value of tasks chosen in the previous transmission.
- **Truncation**, which was originally developed to maximize the transmitted data bit but not the total value of integrally transmitted packets [12]. We modify it and make it work in our scenario by transmitting packets integrally. That is, the packet that was the earliest one executed in the previous transmission will be satisfied first in later transmission.

Recall that our online algorithm HST is actually applicable to the more general non-FIFO tasks. Thus, besides the simulations over FIFO tasks, we will further conduct simulations on non-FIFO tasks to comprehensively validate its performance.

#### 4.2.1. Simulation Results on FIFO Tasks

We introduce the defalt setting of our simulations. We set the energy budget to be E=11,000*J*. The value of h(t) is generated according to a normal distribution N(0.8,1). The schedule determines a rate once per second. In the equation $r\left(t\right)=\frac{1}{2}\log\left(1+h\left(t\right)p\left(t\right)\right)$, the unit of r(t) is KB/S while the unit of p(t) is mW. The number of packets is set as 1500 by default. Packets are generated with their loads randomly sampled from [1,5] and values randomly sampled from [1,10]. The arrival time $r_{i}$ of each packet $J_{i}$ is randomly sampled from $\left[r_{i-1},r_{i-1}+28\right]$ with $r_{0}=0$ and accordingly the deadline $d_{i}$ is randomly sampled from $\left[\max\left\{r_{i},d_{i-1}\right\},\max\left\{r_{i},d_{i-1}\right\}+14\right]$, which guarantees the FIFO property of the generated task set. To be specific, in the following simulations, we choose u=4 as the input of HST if not specified. The default settings of parameters are summarized in Table 2.

**Figure 5:**
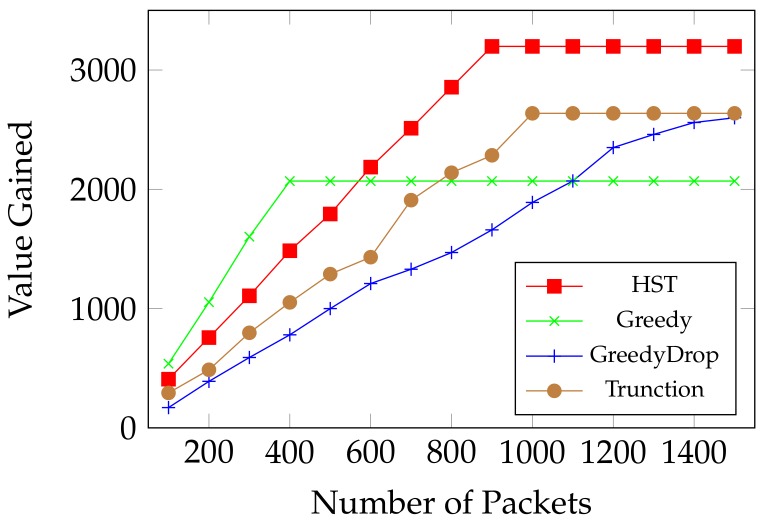
Online MTEF: comparison between HST, Greedy, GreedyDrop and Truncation on FIFO tasks.

We first evaluate the performance of HST when the number of data packets changes. As shown in Figure 5, Greedy has a good performance when there is less packets, because of its greedy nature when energy is sufficient to transmit them all. Both GreedyDrop and Truncation perform better than Greedy when the number of packets increases. Yet all these three algorithms go to ceilings of gained values because they used up the energy. However, by comparing the ceiling values, we notice that our proposed HST algorithm shows up its advantage when more data packets arrive. We can see that HST is more adaptive to harsh environments where not all data packets can be transmitted. This demonstrates the effectiveness of computing a local optimal solution in the algorithm. Moreover, the total value obtained by HST is much higher than other algorithms, which validates the efficiency of HST on maximizing the weighted throughput.

**Figure 6:**
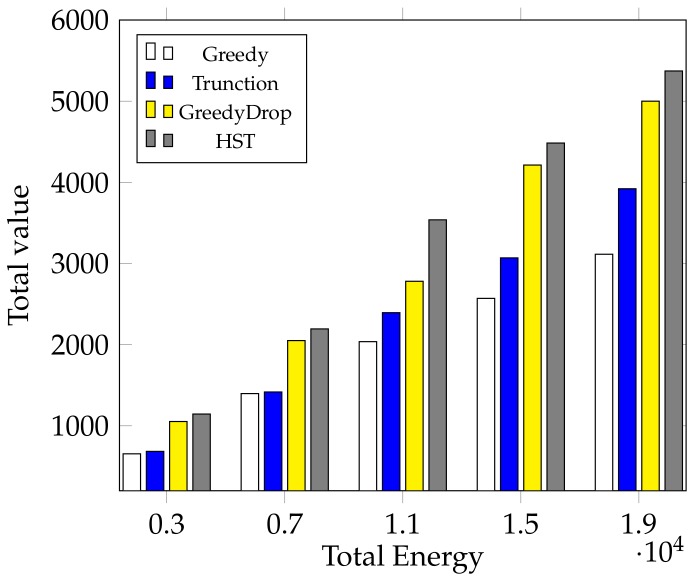
The effect of different amount of energy budget for FIFO tasks.

To further validate the performance of HST, we examine the effect of the amount of energy budget *E*. The energy budget is increased from 3000J to 19000J with a step of 4000. As shown in Figure 6, when *E* grows, HST still outperforms all other algorithms. This validates that HST is efficient under different amount of energy budget.

**Figure 7:**
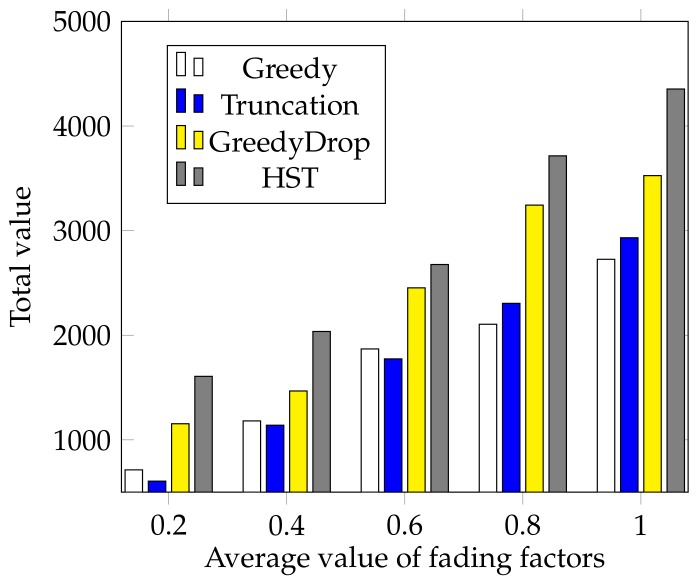
The effect of different average values of fading factors for FIFO tasks.

We further evaluate the performance of HST when the fading factor changes. In this simulation, the fading factors are generated according to a normal distribution N(a,0.025) where *a* varies from 0.2 to 1 with a step of 0.2. We can see from Figure 7 that the total values gained by HST and other algorithms increase as the average fading factor *a* increases. Besides, the total value gained by HST is much higher than that of other algorithms, which validates the efficiency and the robustness of HST again.

**Figure 8:**
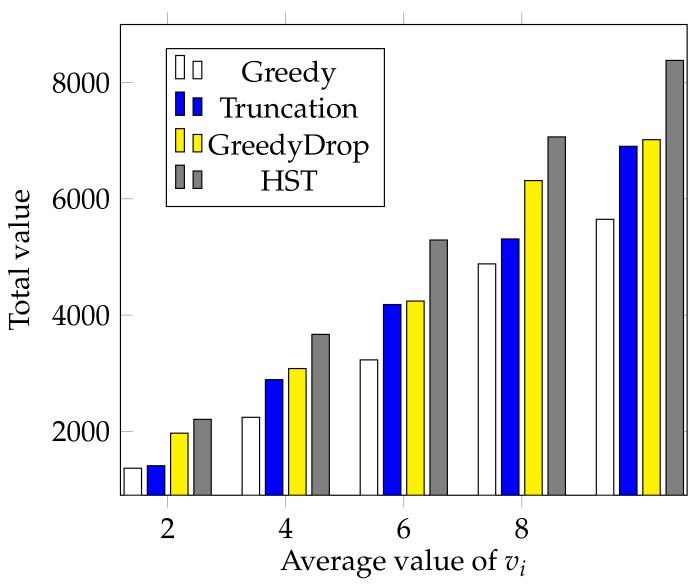
The effect of different average values of $v_{i}$ for FIFO tasks.

We also conduct a simulation to validate the performance of HST when the average value of $v_{i}$ changes. With all other settings the same as the default settings, we change the average value of $v_{i}$ from 2 to 10 with a step of 2. As shown in Figure 8, the total value gained by different algorithms increases almost linearly as the average value of $v_{i}$ grows. The advantage of HST over other algorithms is still apparent when the average value of $v_{i}$ changes. This further validates the HST performs outstandingly under different average values of $v_{i}$.

**Figure 9:**
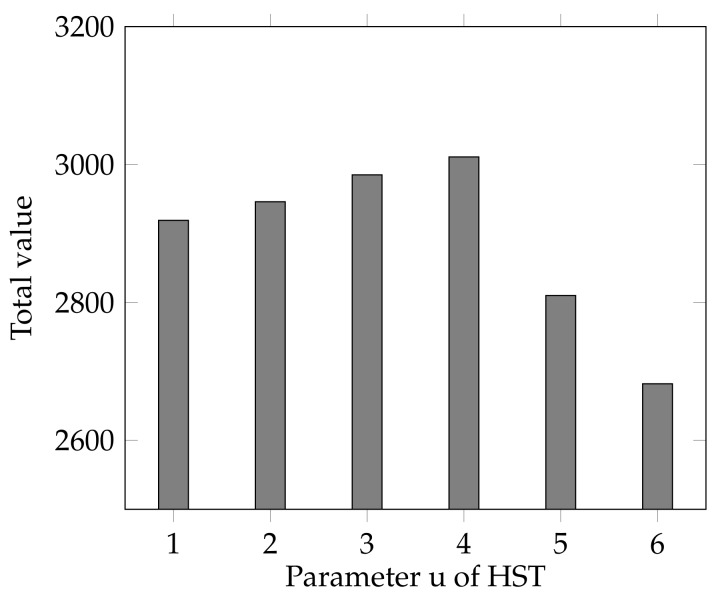
The effect of different value of u.

As parameter *u* is also an important index of HST, we also examine its influence on the results by varying it in range [1,6]. As shown in Figure 9, the highest total value is achieved at u=4. When *u* is too small, e.g., u∈[1,3], each decision is made too early to collect enough information. When *u* is too high, e.g., 5 or 6, the “hesitation” time is too long, which makes the decision too late to respond to the requests early. In practice, the selection of value *u* should be tested and we suggest set an intermediate value that is not too large.

#### 4.2.2. Simulation Results on Non-FIFO Tasks

As mentioned before, HST is capable of handling non-FIFO tasks. Thus, we further conduct simulations on non-FIFO tasks to comprehensively evaluate the performance of HST. In this simultion, we will compare our algorithm HST with Greedy and GreedyDrop, considering that the algorithm Truncation is not applicable to non-FIFO tasks. Note that the default settings of the simulations keep the same as the previous ones if not specified. The difference in the generation of non-FIFO tasks is that deadlines are sampled from $\left[r_{i},r_{i}+14\right]$.

**Figure 10:**
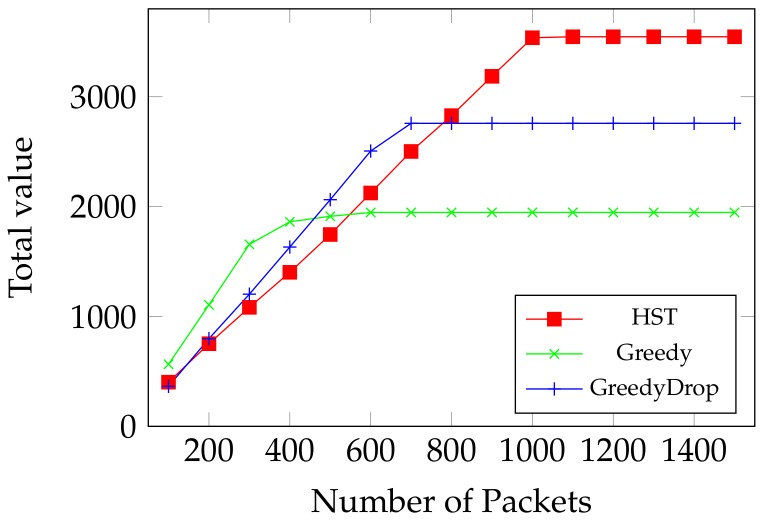
Comparison between HST, Greedy and GreedyDrop when running over non-FIFO tasks.

We first evaluate the performance of HST when the number of packets increases. As shown in Figure 10, although the total value gain by HST is reduced when running over non-FIFO tasks, HST still outperforms other algorithms as more packets arrive. Moreover, while other algorithms goes to the ceiling, the total value gained by HST is much higher than that gained by others.

**Figure 11:**
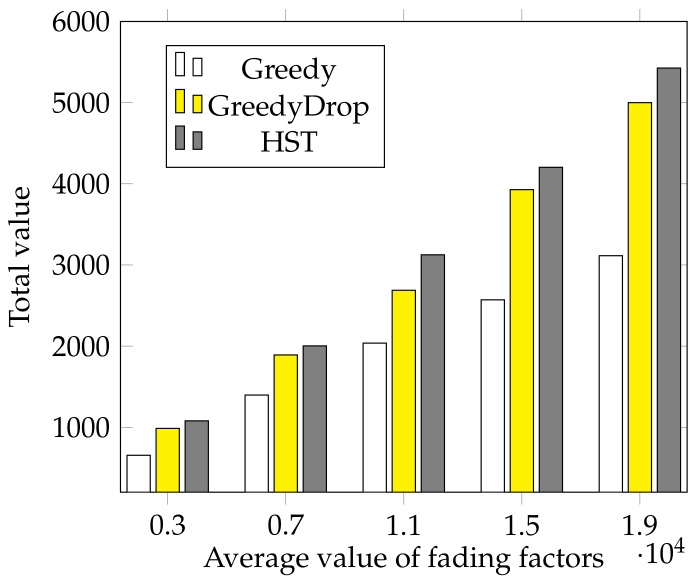
The effect of different amount of energy budget for non-FIFO tasks.

We further examine the effect of the energy budget *E*. As shown in Figure 11, when *E* grows, HST still outperforms Greedy and GreedyDrop for non-FIFO tasks.

**Figure 12:**
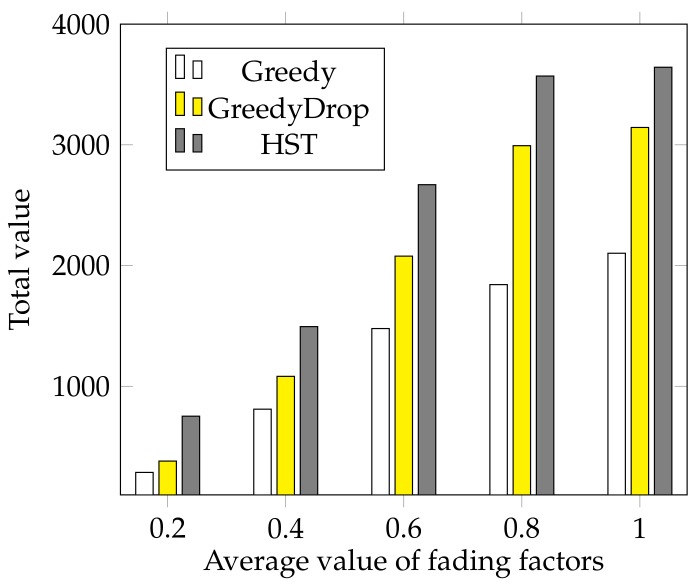
The effect of different average value of $v_{i}$ for non-FIFO tasks.

We also conduct simulations to evaluate the performance under various average values of fading factors on non-FIFO tasks. As shown in Figure 12, the advantage of HST is apparent compared with both Greedy and GreedyDrop when the average fading factor changes.

**Figure 13:**
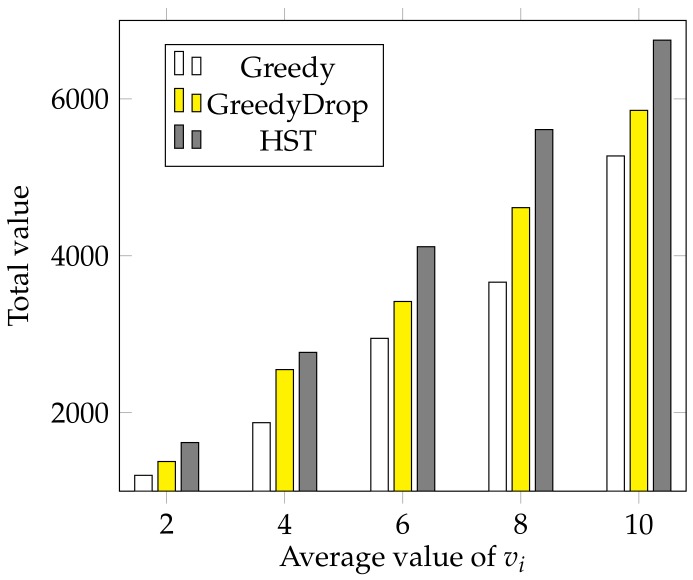
The effect of different average value of fading factors for non-FIFO tasks.

Besides, we conduct a simulation to examine the influence of the average value of $v_{i}$. As we can see from Figure 13, the total value gained by different algorithms increases almost linearly as the average value of $v_{i}$ increases. At the same time, HST still outperforms other algorithms extensively when the average value of $v_{i}$ varies.

The simulation results above further validate the efficiency and the the robustness of our online algorithm when running over non-FIFO tasks.

## 5. Related Work

We review the related work on energy-efficient rate scheduling problems in this section.

In the past decades, many research works have studied the rate-adaptive transmission policies in terms of different objectives, e.g., minimizing the completion time or minimizing the energy consumption [17,18]. For example, Ozel et al. [7] study the completion-time minimization problem in rate scheduling and develop an optimal rate-adaptive transmission policy. Ozel et al. [8] and Yang et al. [9,19], study the problem with the same objective for energy harvesting devices where energy is dynamically acquired from the environment during the scheduling. For the minimization of energy consumption, Prabhakar et al. [3,4] first formulate the energy minimization problem under the deadline constraints of the packets and provide the optimal offline algorithm, but it only works for packets with a common deadline. More complex models where each packet has a respective deadline are discussed in [20,21], but the optimal algorithm can only deal with situations where inter-arrival times of all considered packets are all identically distributed. Finally, Zafer and Modiano [6] extend the packet model to allow general individual deadlines, where earlier arrived packets have no later deadlines, and they develop the optimal rate schedule towards it.

The algorithms above are developed for data transmissions in static channels. In wireless transmission, the channel state is usually unstable, which means sending the same amount of data may need different amounts of energy cost due to the variation of channel status. Thus, the work in [5] consider the fading effect and develop the rate schedule to minimize the energy cost in fading channels. Uysalbiyikoglu et al. [5] extend the work to allow packets to have different arrival time but a common deadline and offer an optimal rate scheduling algorithm towards it. Inspired by [20], Chen et al. [16] solve the special case where all packets have the same available periods. Eventually, Zafer et al. [14] develop the optimal min-energy rate schedule under fading channels for packets that follow FIFO service rule. Shan et al. [22] further extend the result to packets with arbitrary deadlines. The energy minimization problem for energy harvesting devices is also addressed in [23] and more studies concerning energy harvesting devices can be referred to in survey paper [15].

All the works above assume the energy is enough to afford the transmission of all packets. However, such an assumption is not true anymore for battery-capacitated wireless devices. This leads to research works on maximizing the overal transmission throughput under the energy constraint. Fu et al. [10] first develop the optimal algorithm to maximize the transmitted data bits before a given deadline with constrained energy in the static channel. Shan et al. [12] consider packets that have individual deadlines and follow FIFO service rule, and they develop the optimal algorithm to maximize the transmitted data bits. Ahmed et al. [11] and Fu et al. [10] develop algorithms under fading channels to maximize the transmitted data bits with a given energy budget. Ozel et al. [8] and Wu et al. [13] further consider energy harvesting devices and develop offline and online competitive algorithms to maximize the transmitted data bits under dynamical energy arrivals. All these solutions on maximizing the transmitted data bits, however, cannot be applied to scenarios that data packets may have individual delay constraints and only fully transmitted packet can contribute a value, which is practical in many applications.

Therefore, it is desirable to develop max-throughput packet transmission policies under the energy and integrity constraints that packets are required to be integrally transmitted, which is merely studied. This paper addresses such a problem by designing the optimal algorithm and heuristic algorithms.

## 6. Conclusions

For battery-capacitated wireless devices, data packets cannot always be completely transmitted in wireless data communication. Noting that partially transmitted packets may contribute nothing to the overall throughput, we introduce the measure of weighted throughput, i.e., the total value of integrally transmitted data packets. This paper addresses the energy-efficient rate scheduling problem over fading channels to maximize the weighted throughput under the energy constraint. In the offline setting, we design an optimal algorithm that runs in pseudo-polynomial time to maximize the weighted throughput of transmitted packets, which is the best possible solution considering that the problem is NP-hard. In the online scenario, we design an algorithm by calling the derived local optimal solution. Simulation results shows the efficiency of the proposed algorithms in maximizing the throughput. In the further work, it is interesting to investigate effect of energy dynamics to the weighted throughput, e.g., the energy harvesting and wireless charging. It is also worth studying other variants of the problem in the future, e.g., the multiple access scenario.

## Figures and Tables

**Table 1:** Notations.

Symbol	Semantics
J	the set of packets needed transmission
$J_{i}$	*i*th packet
$r_{i}$	arrival time of $J_{i}$
$d_{i}$	deadline of $J_{i}$
$l_{i}$	workload of $J_{i}$
$v_{i}$	value of $J_{i}$
*T*	latest deadline in *J*
r(t)	data transmission rate specified in time *t*
p(t)	energy allocated in time *t*
h(t)	fading factor in time *t*
$x_{i}$	a binary variable representing whether a task is chosen or not
$r_{it}$	the data amount of $J_{i}$ that is transmitted in time slot *t*
w(t)	water level specified in time *t*
*E*	total amount of energy available for data transmission

**Table 2:** Simulation default settings on FIFO tasks.

Symbol	Semantics
$r_{i}$	randomly sampled from $\left[r_{i-1},r_{i-1}+28\right]$
$d_{i}$	randomly sampled from $\left[\max\left\{r_{i},d_{i-1}\right\},\max\left\{r_{i},d_{i-1}\right\}+14\right]$
$l_{i}$	randomly sampled from [1,5]
$v_{i}$	randomly sampled from [1,10]
h(t)	generated according to a normal distribution N(0.8,1)
*E*	11,000*J*
Number of packets	1500

## Appendix A

**Introduction to the concept of water level:** We give a short introduction to the concept of water level by examining a simple scenario (simplified from [8]) where a transmitter aims to transmit as much data bits as possible using a limited energy *E*. Since the fading function h(t) is a piecewise constant function, we assume there are *m* pieces/intervals in the fading function. The length of the *l*th interval is $L_{l}$ and the energy allocated per time slot in that period is $p_{l}$. According to Equation (1), the optimization problem under such a simple scenario is formulated as follows,

(A1) $$\begin{array}{cc} max\; & \sum_{l=1}^{m}\frac{L_{l}}{2}\log\left(1+h_{l}p_{l}\right) \end{array}$$

(A2) $$\begin{array}{cc} s.t.\; & \sum_{l=1}^{m}L_{l}p_{l}\leq E \end{array}$$

(A3) $$\begin{array}{c} p_{l}\geq 0 \end{array}$$

This optimization problem is a concave optimization problem with a unique maximum value. Hence, we can construct the following Lagrangian function by introducing variants λ and $\mu_{l},l=1,2,...,m$.

(A4) $$\Phi =\sum_{l=1}^{m}\frac{L_{l}}{2}\log\left(1+h_{l}p_{l}\right)-\lambda \left(\sum_{l=1}^{m}L_{l}p_{l}-E\right)-\sum_{l=1}^{m}\mu_{l}p_{l}$$

We have the complimentary slackness conditions as below.

(A5) $$\begin{array}{c} \lambda (\sum_{l=1}^{m}L_{l}p_{l}-E)=0 \end{array}$$

(A6) $$\begin{array}{c} \mu_{l}p_{l}=0,\forall l\in \left[1,m\right] \end{array}$$

By the necessary and sufficient KKT conditions, we have $\frac{\partial \Phi }{\partial p_{l}}=0$ and $u_{l}p_{l}=0$. Thus,

$$\frac{L_{l}}{2}\frac{h_{l}}{1+h_{l}p_{l}}-\lambda L_{l}=0,\forall l\in \left[1,m\right].$$

Hence, the objective function achieves a maximum value when

(A7) $$p_{l}={\left[\frac{1}{2\lambda }-\frac{1}{h_{l}}\right]}^{+},\forall l\in \left[1,m\right]$$

Accordingly, we can define a notion of water level $w=p_{l}+\frac{1}{h_{l}}$ as introduced by [8]. In ref. [8], the authors take the energy as water and propose a water-filling scheme to solve the optimization problem addressed therein. If *E* units of energy/water is filled in a rectangle with a length *L*, the resulting water level would be $\frac{E}{L}$. In general, if p(t) units of energy is allocated to time slot *t*, the resulting water level w(t) at that time would be p(t) plus a constant $\frac{1}{h(t)}$, the multiplicative inverse of the fading factor h(t) at time slot *t*. The optimization problem is then equivalent to allocating the water/energy to a sink with an uneven bottom (where the *l*-th part has a height $\frac{1}{h_{l}}$). Therefore, the computation of the optimal energy allocation is transformed to that of computing the optimal water level.

Different from the simple scenario above, the MTEF problem addressed in this paper further considers the respective time constraints of packets and the integrity constraints that the chosen packets should be integrally transmitted, which is much more complicated.
